# Environmental DNA reveals the fine-grained and hierarchical spatial structure of kelp forest fish communities

**DOI:** 10.1038/s41598-021-93859-5

**Published:** 2021-07-14

**Authors:** Thomas Lamy, Kathleen J. Pitz, Francisco P. Chavez, Christie E. Yorke, Robert J. Miller

**Affiliations:** 1 Marine Science Institute, University of California, Santa Barbara, CA 93106 USA; 2 MARBEC, University of Montpellier, CNRS, Ifremer, IRD, Sète, France; 3 Monterey Bay Aquarium Research Institute, Moss Landing, CA 95039 USA

**Keywords:** Biodiversity, Community ecology, Conservation biology, Molecular ecology

## Abstract

Biodiversity is changing at an accelerating rate at both local and regional scales. Beta diversity, which quantifies species turnover between these two scales, is emerging as a key driver of ecosystem function that can inform spatial conservation. Yet measuring biodiversity remains a major challenge, especially in aquatic ecosystems. Decoding environmental DNA (eDNA) left behind by organisms offers the possibility of detecting species sans direct observation, a Rosetta Stone for biodiversity. While eDNA has proven useful to illuminate diversity in aquatic ecosystems, its utility for measuring beta diversity over spatial scales small enough to be relevant to conservation purposes is poorly known. Here we tested how eDNA performs relative to underwater visual census (UVC) to evaluate beta diversity of marine communities. We paired UVC with 12S eDNA metabarcoding and used a spatially structured hierarchical sampling design to assess key spatial metrics of fish communities on temperate rocky reefs in southern California. eDNA provided a more-detailed picture of the main sources of spatial variation in both taxonomic richness and community turnover, which primarily arose due to strong species filtering within and among rocky reefs. As expected, eDNA detected more taxa at the regional scale (69 vs. 38) which accumulated quickly with space and plateaued at only ~ 11 samples. Conversely, the discovery rate of new taxa was slower with no sign of saturation for UVC. Based on historical records in the region (2000–2018) we found that 6.9 times more UVC samples would be required to detect 50 taxa compared to eDNA. Our results show that eDNA metabarcoding can outperform diver counts to capture the spatial patterns in biodiversity at fine scales with less field effort and more power than traditional methods, supporting the notion that eDNA is a critical scientific tool for detecting biodiversity changes in aquatic ecosystems.

## Introduction

Two centuries ago the influential naturalist Comte de Buffon noted that “*the entire face of the earth bears the imprint of human power*”. Today these changes have only accelerated, as humans are driving worldwide deterioration of nature and declines in biodiversity^1^, risking the collapse of ecosystems and loss of the natural goods and services society depends on^2,3^. Underlying these declines are complex patterns of changing species composition as habitat loss and climate change interact to drive species range shifts and extinctions^4,5^. To assess how changes in biodiversity scale from local (α) to regional (γ) scales, and thus inform conservation efforts, ecologists have used measures of beta (β) diversity^6–8^, a spatial component of biodiversity which reflects the interplay between fundamental processes controlling the relative abundances of species across landscapes, such as dispersal, habitat preferences and ecological drift^9^.

Evidence is growing that the multiscale nature of biodiversity, particularly β diversity, has a crucial role in maintaining ecosystem functions, although most research to date has focused mostly on α diversity^8,10^. Biotic homogenization, in particular, arises through species introductions, extinctions, and human transformation of landscapes, a mixture of processes termed the “*anthropogenic blender*” by Olden^11^. Measured as declines in β diversity, biotic homogenization can negatively affect ecosystem function as strongly or more so than α diversity^12^. Furthermore, β diversity declines^13^ can be accompanied by increasing α diversity due to species introductions^14^, obscuring overall negative impact on ecosystem multifunctionality^8,11^. Because different species assemblages are required to perform the diverse set of ecosystem functions that define natural heterogeneous landscapes, sustaining them requires high β diversity^15^.

Despite its importance, accurately assessing biodiversity remains one of the greatest challenges ecologists face^16^. This is especially true in aquatic ecosystems that host a vast reservoir of species but are challenging to observe. Fishes are the most prevalent vertebrates in aquatic ecosystems, but are difficult to survey using traditional methods due to their often sparse density, cryptic nature, mobility and elusive behavior^17,18^. Fish support key ecosystem services, especially in coastal areas where millions of people depend on them for food and income, but their populations are increasingly vulnerable to overfishing, introduced species, pollution, and habitat loss^19,20^. Here we explore a novel way of measuring β diversity in marine fish communities using environmental DNA and compare it to traditional visual long-term surveys.

Environmental DNA (eDNA) is shed by aquatic organisms via many sources including fecal matter, mucus, or skin, forming a suspended biodiversity database. Sampling eDNA and sequencing it using high-throughput methods can reveal the presence of a wide spectrum of species without direct observations or sampling of whole organisms^21–24^. In aquatic ecosystems, eDNA metabarcoding of water samples may detect more species and avoid some sources of bias inherent to traditional surveys^25–29^.

While eDNA has been successfully used to capture regional diversity in a variety of systems^25–29^, recent studies have revealed that eDNA can also be used to describe spatial patterns of biodiversity and species composition at relatively fine scales (< 1 km) across natural marine landscapes^27,29–33^. These results suggest that eDNA could be of great utility for measuring β diversity over spatial scales small enough to be relevant to ecosystem function and conservation.

**Figure 1:**
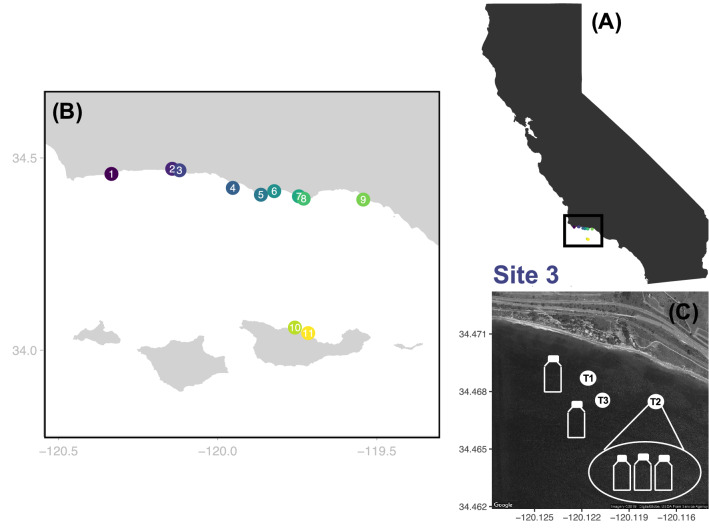
Hierarchical distribution of sampling within the Santa Barbara Channel (SBC), California. (**A**) Location of the study area along the California coastline. (**B**) Distribution of the 11 rocky reef sites within the SBC. Fish communities were visually surveyed at two to three transects within each site (N = 27 transects). Triplicate water samples were collected within one transect and single water samples were collected in the remaining transects for eDNA metabarcoding (N = 49 bottles). (**C**) Close up of site 3 where fish communities were visually surveyed and eDNA sampled at three transects. Triplicate water samples were collected at transect 2 and single water samples were collected at transects 1 and 3.

Here we used information from eDNA metabarcoding of the hypervariable region of the *12S* mitochondrial rRNA to capture β diversity of fish communities on temperate rocky reefs hosting underwater forests of the giant kelp *Macrocystis pyrifera*, a foundation species that creates complex structure, is highly productive, and harbors a diverse community of reef organisms^34,35^. Kelp forest fish communities are highly dynamic and influenced by a range of environmental factors at multiple spatial scales^36^, as well as by fishing and ocean warming^37,38^. Traditional surveys of these communities rely on divers counting fish in defined areas, and are inherently biased towards conspicuous and bold species and limited by underwater logistics and visibility. We coupled a detailed survey of bony (*Actinopteri*) and cartilaginous (*Chondrichthyes*) fishes based on both standard diver-executed Underwater Visual Censuses (UVC) and eDNA metabarcoding in a spatially structured hierarchical sampling design of 27 transects distributed across 11 rocky reef sites to investigate β diversity of fish communities in the Santa Barbara Channel in Southern California (Fig. 1). We assessed the main sources of β diversity by (i) directly partitioning regional γ diversity and (ii) investigating spatial variation in multivariate community structure across spatial scales^6^. Our results demonstrate that eDNA can be used to measure and capture the main source of β diversity of marine fish communities with broader taxonomic resolution and much less field effort than traditional methods, portending an important role for eDNA in biodiversity monitoring in the twenty-first century.

## Results

**Figure 2:**
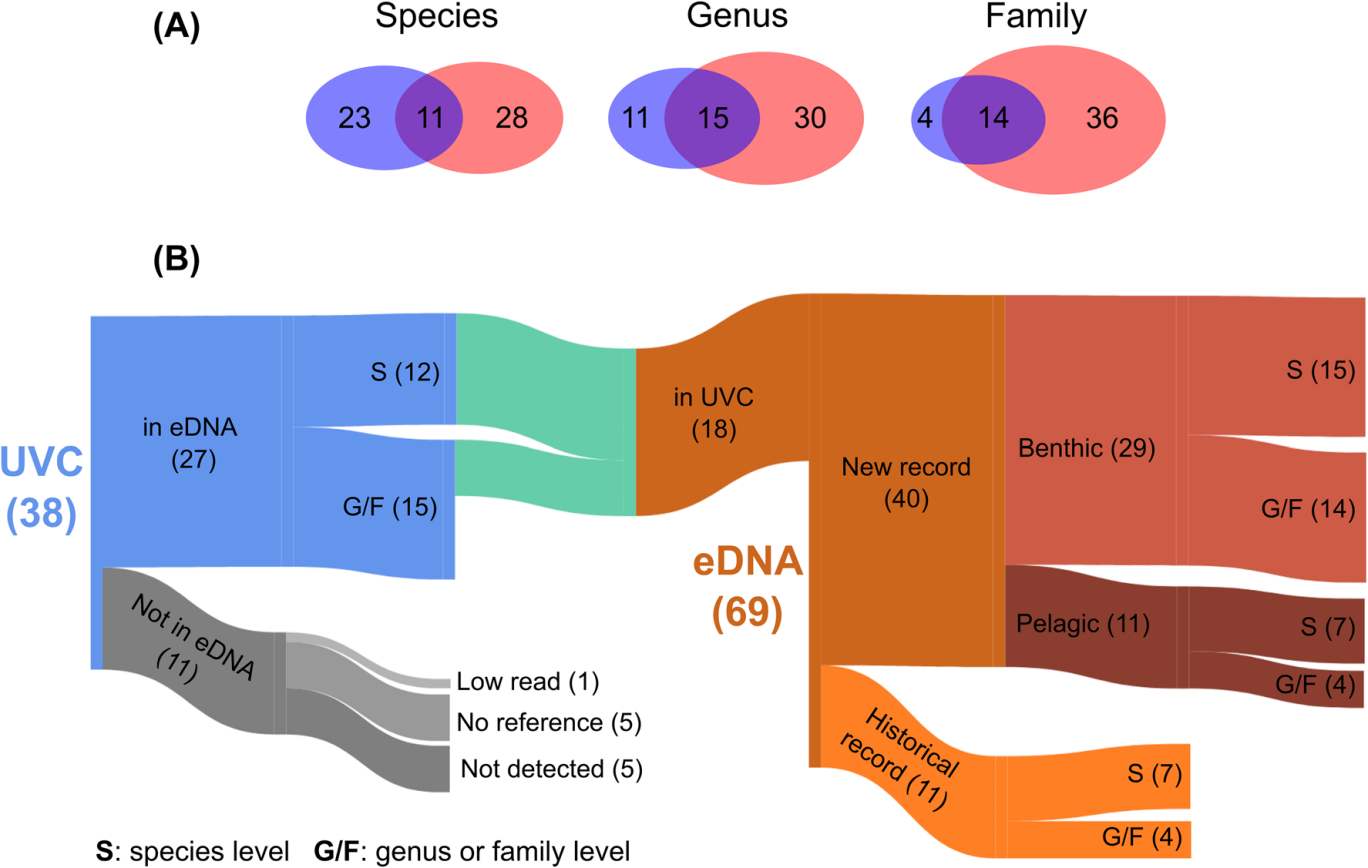
(**A**) Venn diagrams showing the overlap of unique fish species, genera and families detected by Underwater Visual Census (UVC: blue) and eDNA metabarcoding (red). (**B**) Sankey diagram illustrating the relationships between the 38 fish taxa detected using UVC and the 69 fish taxa detected using eDNA metabarcoding. Bar width is proportional to the number of taxa. Note that due to discrepancy in taxonomic resolution, 27 fish taxa that were visually detected were lumped into 18 taxa to match taxa from eDNA metabarcoding.

Overall, eDNA metabarcoding identified nearly double the taxa recorded from diver visual surveys (${\gamma }_{eDNA}$ = 69 and ${\gamma }_{UVC}$ = 38). We visually detected 38 unique fish taxa at the regional scale, including 34 species, two genera and two families (Supplementary Table SM1). Using eDNA metabarcoding, we characterized 69 unique taxa at the regional scale corresponding to 41 species, 8 genera and 20 families (Supplementary Tables SM1 and SM2). At the species level we detected a similar number of taxa with both methods, with 11 species detected by both methods (Fig. 2). Yet when compared at the genus and family levels, eDNA metabarcoding identified many more unique taxa: 50 unique fish families were detected by eDNA while only 18 were evident in the visual surveys, of which 14 families were jointly detected by both methods (Fig. 2). Six eDNA-detected families not present in the visual survey have been previously recorded in the region from 2000 to 2018 (N = 748 transects).

To further compare visual surveys (UVC) and eDNA metabarcoding, we collapsed UVC taxa into genera and families to match the taxonomic resolution of eDNA metabarcoding (Supplementary Table SM1; Fig. 2). For example, we visually detected nine species of the diverse genus *Sebastes* (rockfishes), which could not be distinguished at the species level by our *12S* rRNA sequence. However, when lumped at the genus level both methods detected rockfishes in similarly high numbers across samples. This resulted in a final 18 taxa jointly detected by both methods, for which the number of eDNA sequence reads was positively related to the observed biomass (*R*^2^ = 0.225, *P* = 0.047; Supplementary Fig. SM1). Both methods detected some of the most characteristic giant kelp forest species in great numbers (i.e., reads or biomass), including señorita (*Oxyjulis californica*), black surfperch (*Embiotoca jacksoni*), sheephead (*Semicossyphus pulcher*), blacksmith (*Chromis punctipinnis*), pile perch (*Rhacochilus vacca*), halfmoon (*Medialuna californiensis*) and garibaldi (*Hypsypops rubicundus*) (Fig. 2 and Supplementary Fig. SM1, Supplementary Table SM1). Eleven taxa that were not detected using eDNA included one that was discarded due to a small number of reads (*Porichthys notatus*), five taxa that had no sequence available in the reference database (*Phanerodon furcatus*, *Anisotremus davidsonii*, *Alloclinus holder*, *Rhacochilus toxotes* and *Bothidae*), and five taxa that eDNA failed to detect despite being visually detected and represented in the library (Supplementary Table SM1). These taxa contributed little to the biomass observed on each reef (Supplementary Figs. SM2 and 3, Supplementary Table SM1), with the exception of the kelp bass (*Paralabrax clathratus*), a large and common fish across samples.

51 taxa were detected only by eDNA (Fig. 2 and Supplementary Fig. SM4, Supplementary Table SM2), comprising 29 species, 5 genera and 17 families. 11 were previously detected in the region from 2000 to 2018, including giant kelpfish (*Heterostichus* spp.), lingcod (*Ophiodon elongatus*), and four elasmobranchs (leopard shark *Triakis semifasciata*, horn shark *Heterodontus francisci*, bat ray *Myliobatis californica* and Scyliorhinidae). eDNA also identified many pelagic species typically not recorded by visual surveys in the region, such as Japanese pilchard (*Sardinops melanostictus*), anchovies (*Engraulis* spp.), mackerels (*Trachurus* spp. and *Scomber* spp.), topsmelts (*Atherinopsidae*) and great white shark (*Carcharodon carcharias*). Finally, eDNA detected 29 benthic taxa that were never visually recorded before, including numerous reads of the cryptic benthic family *Gobiesocidae*, and species from adjacent sandy habitat including *Sciaenidae*, barred surfperch (*Amphistichus argenteus*) and the California lizardfish (*Synodus lucioceps*).

**Figure 3:**
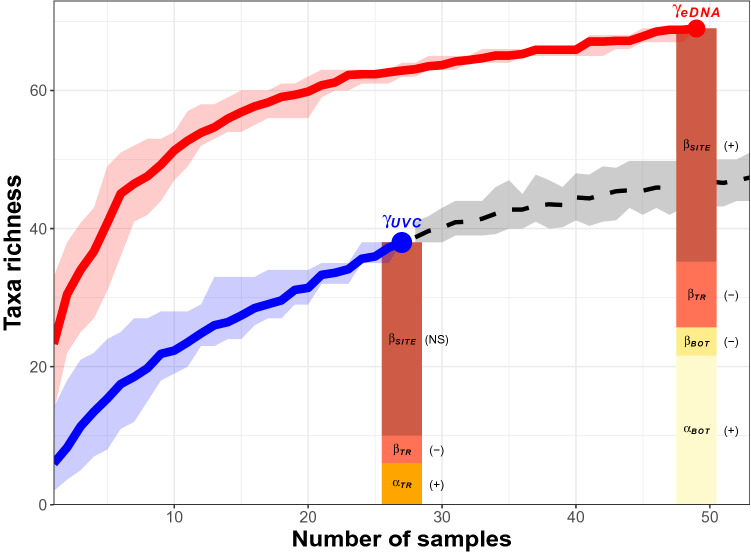
Spatial variation in fish taxonomic richness. Taxa Area Relationships for eDNA (red line) and UVC (blue line) along their 95% confidence intervals. The dashed black extension of the UVC line estimates how many more taxa could have been detected using UVC if more transects were sampled based on random draws from a pool of 748 transect surveys from 2000 to 2018. Bar plots represent the additive partitioning of regional taxonomic richness among sources of variation for eDNA (${\gamma }_{eDNA}$) and UVC (${\gamma }_{UVC}$). Significance levels: higher than expected (+), lower than expected (−) and non-significant (NS) component of variation.

Regional taxonomic richness estimated from eDNA (${\gamma }_{eDNA}$ = 69) exceeded its UVC counterpart (${\gamma }_{UVC}$ = 38; Fig. 3). However, in both cases, regional taxonomic richness was primarily driven by (i) higher taxonomic richness in the smallest sampling unit, either individual water samples for eDNA (${\alpha }_{BOT}$ = 21.59, *P* < 0.001) or individual transects for UVC (${\alpha }_{TR}$ = 6, *P* < 0.001; Fig. 3) and (ii) high spatial variation among sites (${\beta }_{SITE}$ = 33.82, *P* < 0.001 for eDNA and ${\beta }_{SITE}$ = 28, *P* = 0.135 for UVC; Fig. 3B). Importantly, variation among triplicate bottles within transects was smaller than expected (${\beta }_{Bottle}$ = 4.11, *P* < 0.001; Fig. 3B) and contributed little to eDNA regional taxonomic richness. The spatial variation among transects within each site was also smaller than expected both for eDNA (${\beta }_{TR}$ = 9.48, *P* = 0.001) and UVC (${\beta }_{TR}$ = 4, *P* = 0.001). Significantly lower values of ${\beta }_{Bottle}$ and ${\beta }_{TR}$ indicate the similarity of the taxa detected at these two scales, revealing the ability of eDNA to capture the diversity of each transect. Conversely, significantly higher values of eDNA-based ${\alpha }_{BOT}$ and ${\beta }_{SITE}$ indicate the non-random organization of taxa across sites and demonstrate the ability of eDNA to detect species sorting across sites, which were not evident based on UVC. Examination of the Taxa-Area Relationships (TAR) (Fig. 3) also indicates very distinct patterns of taxa accumulation with space. The eDNA TAR initially accumulated quickly with space (2.76 × S) before plateauing at 11.37 samples (95% CI [10.59–12.14], 0.36 × S after this point). In contrast, discovery rate of new taxa was exponential for UVC (6.47 × S^0.53^), with no sign of saturation, suggesting that 27 samples were not nearly enough to estimate regional taxonomic richness. To investigate what the UVC curve might look like beyond 27 samples we randomly selected additional UVC samples from the historical collection of samples from 2000 to 2018 (Supplementary Fig. SM5). Our result suggests that 6.9 times more UVC samples would be required to detect 50 taxa compared to eDNA (69 vs. 10 samples).

**Figure 4:**
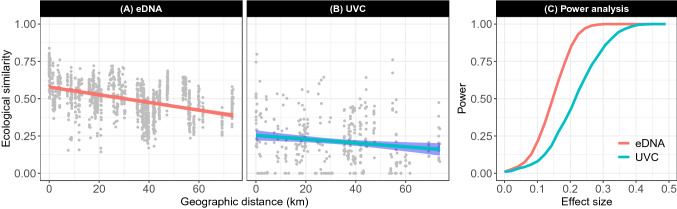
Decay of ecological similarity with geographic distance. Ecological similarity was measured as (1 − *D*) with *D* the Bray–Curtis dissimilarity index between pairs of eDNA samples (**A**) or UVC samples (**B**). Each line represents 95% confidence intervals. (**C**) Power curves of distance-decay relationship at a significance level of 0.05 for eDNA and UVC.

**Figure 5:**
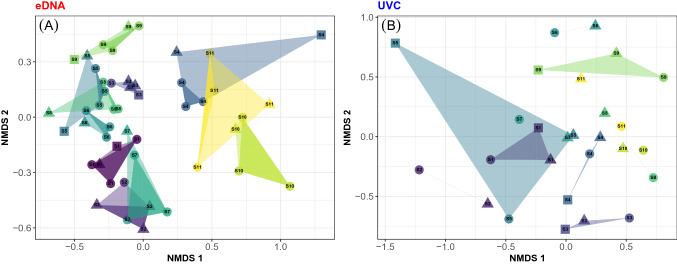
Spatial variation in community structure among samples based on Non-Metric Multidimensional Scaling (NMDS) ordinations for eDNA (**A**) and UVC (**B**). Samples are colored by site (light colored hulls) and triplicate water samples (in **A**) are colored using darker hulls. Transects within sites are represented using different symbols. S1 to S11 indicates site number. Color code is as in Fig. 1. Ordination are based on Bray–Curtis dissimilarities of the square root number of reads (**A**) and observed biomass (**B**) of fish taxa. Taxa scores are showed in Supplementary Fig. SM6.

Investigation of the spatial variation in community structure (compositional beta diversity) based on the Bray–Curtis dissimilarities among samples revealed very similar patterns. First, hierarchical partitioning of the variation also showed that the main source of variation occurred among rocky reef sites for both methods (UVC 33.4% and eDNA 60.0%), while variation among both transects (UVC 1.1% and eDNA 0.2%) and triplicate water bottles (eDNA − 0.2%) were very small. Second, the ecological similarity between pairs of samples is expected to decrease with increasing geographical distance^39^. This distance-decay relationship fit much more strongly to eDNA data (Mantel test: *r* = 0.426, *P* < 0.001) compared to UVC data (*r* = 0.149, *P* = 0.045; Fig. 4). eDNA exhibited higher power to detect significant spatial structure based on the Mantel test (Fig. 4). For example, eDNA had 94% more power than UVC to detect a spatial correlation of 0.2 at a significance level of 0.05 (0.86 vs. 0.44; Fig. 4). Taken from the estimated intercept of a linear fit of the distance-decay relationships, the ecological similarity between replicate bottles taken from the same transect for eDNA was 0.58 versus 0.25 for UVC data, indicating higher reproducibility in eDNA sampling versus diver censuses. Slopes of these relationships, furthermore, are direct measures of rate of change in community structure with space. The slope for eDNA (− 0.0026 km^−1^) was twice as steep as that for UVC (− 0.0012 km^−1^) showing a higher rate of change in eDNA data with respect to geographic distance. These results were further illustrated in the NMDS ordination displaying variation in community structure among samples in two dimensions. The ordination shows the overall similarity between triplicate bottles that form clusters of samples with each site in contrast to the large compositional variation among the 11 sites, which mostly form distinct clusters (Fig. 5). For instance, the two Channel Island sites (10 and 11) emerge as compositionally very distinct from the mainland sites (1–9). This result agrees with previous studies showing important differences in the environmental conditions and fish community structure between mainland and island sites^36,40^. The NMDS ordination based on UVC data provided a less clear picture of the spatial structure of the fish communities, with less pronounced differences and greater overlap between sites. Indicator species analysis based on eDNA revealed that nine taxa were specific to a given rocky reef site while the same analysis based on UVC data resolved only two indicator taxa (Table 1).

**Table 1:** List of significant indicator taxa across the 11 sites. For each taxon we provide its indicator value (**indcls**), the site in which it is more prevalent and adjusted probability of obtaining as high an indicator value as observed over 1000 iterations.

Taxa	Common name	SITE	indcls	P-value
**eDNA**				
*Sardinops melanostictus*	Japanese pilchard	2	0.16815	0.0198
Gobiesocidae	Clingfishes	3	0.28686	0.0198
*Heterodontus francisci*	Horn shark	5	0.6211	0.0198
*Triakis semifasciata*	Leopard shark	7	0.47448	0.0069
*Notorynchus cepedianus*	Broadnose sevengill shark	7	0.74169	0.0069
*Amphistichus argenteus*	Barred surfperch	8	0.25408	0.0372
*Chromis punctipinnis*	Blacksmith	10	0.47518	0.0198
*Medialuna californiensis*	Halfmoon	10	0.55373	0.0069
Sphyraenidae	Barracuda	10	0.48503	0.0488
**UVC**				
*Hypsypops rubicundus*	Garibaldi	6	0.764	0.041
*Lythrypnus dalli*	Bluebanded goby	6	1.000	0.019

## Discussion

Decoding the information contained in suspended environmental DNA was a more powerful approach to measuring β diversity and capturing the main sources of spatial structure in marine kelp forest fish communities than standard underwater visual surveys (UVC). Although only 59% of the taxa detected using eDNA metabarcoding were identified at the species level, the overall information recovered provided a comprehensive understanding of the complex multiscale spatial variation of fish communities across a marine landscape of temperate rocky reefs. Surprisingly, eDNA data detected spatial variation at finer resolution than UVC, capturing strong spatial turnover in both taxonomic richness and community structure among rocky reefs in the Santa Barbara Channel.

Environmental DNA metabarcoding is emerging as an powerful tool to estimate biodiversity in aquatic ecosystems^41^, as numerous studies have shown the great potential of this approach to identify a wide variety of fish^27,30,32,42^. Until recently, doubts remained about the ability of eDNA to detect fine scale spatial structure in marine environments that were generally considered highly connected via water flow^43^. Nevertheless, pioneering work in Monterey Bay, California, demonstrated the potential of eDNA to describe spatial patterns of marine community structure at scales of a few kilometers^27^ and more recent studies have confirmed this in other regions^29–33^. Similarly, our results, from transects a few meters apart within rocky reefs, and rocky reefs ranging from 2 to 74 km apart, show that eDNA metabarcoding can effectively detect relatively fine-scale spatial structure in fish communities and provide higher resolution estimates of β diversity than traditional visual surveys. The Port et al*.* study^27^ also characterized spatial structure along a cross-shore transect, resolving communities separated by ~ 60 m across habitat types comprising seagrass, kelp forest, sandy bottom, rocky reef and open water in a relatively quiescent protected area^44^. In contrast, all our sites were nearshore rocky reefs and kelp forests that were more exposed to water currents^44^, a combination that would be expected to limit the spatial differences in eDNA. Instead, our findings strongly reinforce the conclusion that eDNA can discriminate communities at fine spatial scales as first established by Port et al.^27^, even within habitats.

Fish abundance estimated from eDNA data depends on several factors, including eDNA shedding, degradation and dispersion^45–48^. The persistence of eDNA can vary among aquatic organisms, but a growing body of evidence suggest that eDNA decay rates are faster in marine compared to freshwater environments and that once shed eDNA may only be detectable for hours to a few days^46–48^. For instance, both Andruszkiewicz et al.^47^ and Collins et al.^46^ compiled previous findings on eDNA half-lives, showing they were relatively short in temperate marine environments, ranging from 6.9 h for the northern anchovy^45^ to 63 h for the Maugean skate^49^. Another study found similar eDNA decay rates in anchovy, sardine, and mackerel, with first-order rate constants on the order of 10^−1^ per hour^45^. This corresponds to decay to near or below detection limits within 3–4 days for high equilibrium eDNA concentrations reached after 17–25 h in tank experiments^45^. Based on estimated inshore eDNA half-life and naturally occurring eDNA concentrations, Collins et al.^46^ concluded that eDNA may be detectable for around 2 days. In our natural scenario, therefore, eDNA persistence might be 2–4 days. Fram et al.^50^, however, estimated a mean seawater residence time of 1.1 h for a typical kelp forest off Santa Barbara, an order of magnitude larger than the fish eDNA decay rate measured by Sassoubre et al.^45^. This would suggest that all fish eDNA would be flushed out of the forest every hour. Our results, however, showed high within-transect similarity and greater between-transect variability within kelp forest sites, suggesting that seawater residence time in the forest is longer than Fram et al.^50^ estimated, or that eDNA generation rates are also very high. Residence times in a larger kelp forest off San Diego were estimated to be as long as 7 days^51^, and in any case water flow velocities 1 m above the seafloor, where our samples were collected, are likely much slower than velocities closer to the surface due to boundary effects^52^. Furthermore, there is likely high within-forest variability in water residence time due to bottom topography^53^ and kelp density^54^. Landscape-scale maps of water mixing would be valuable for more detailed interpretation of eDNA data in future studies, although our results suggest that homogenization through water mixing is not a barrier to understanding spatial patterns of kelp forest fish communities.

Our results strongly reinforce the efficacy of eDNA for measuring biodiversity, but the ability to estimate biomass of species would significantly enhance eDNA’s utility as a monitoring and management tool. We found a significant, albeit weak, relationship between the number of sequence reads and the biomass (log scale) of the 18 taxa jointly detected by eDNA and UVC (Supplementary Fig. S1). For instance, the California Sheephead (*Semicossyphus pulcher*) displayed both the highest biomass (245 g) and number of reads (290 reads) per transect. The low explanatory power of variability in this the relationship (r^2^ = 0.22) could be interpreted as indicating large species-specific differences in eDNA shedding or decay rate. However, the high mobility of fishes and consequent limitations of a snapshot UVC survey in estimating their biomass also likely contributes. These are encouraging results linking prevalence of eDNA to fish biomass but still great caution is required when linking species biomass with eDNA reads^48^. Further investigation of sampling and methodological approaches to improve this relationship would be extremely valuable.

The Santa Barbara Coastal Long Term Ecological Research program (SBC LTER) has a nearly 20-year time series of fish community data in the Santa Barbara Channel to compare to our snapshot of eDNA metabarcoding results. Interestingly, eDNA metabarcoding detected taxa never recorded since the time series began in 2000. For instance, we detected nine reads of great white shark at a single site (9: Carpinteria) where juvenile great white sharks are known to occur, are often recorded via acoustic tags and visual sightings, and were also detected in another study using a species-specific eDNA assay^55^, but are highly mobile, making their detection by divers unlikely. Barred surfperch and California lizardfish, species characteristic of adjacent sandy bottom habitats, were also not previously recorded by SBC LTER, and some species previously recorded by SBC LTER were only detected in this sampling event by eDNA metabarcoding, including the less common and highly mobile elasmobranchs leopard sharks and bat rays. eDNA was therefore particularly useful to detect species that are otherwise difficult to see in kelp forest habitats due to elusive behaviors and ability to quickly avoid divers. Our detection of seven Chondrichthyes is particularly interesting given that MiFish—U primer set is designed to target primarily bony fishes^56^, while another primer set, Mifish—E was specifically designed for Chondrichthyes. Amplification through both primer sets could maximize our overall detections.

Surprisingly, we found relatively small overlap in taxa identified by both visual and eDNA metabarcoding surveys, with only 11 species detected by both surveys. This disparity is in part due to the inability of our target amplified region to adequately discriminate between closely related species. For instance, the recent evolutionary radiation of rockfishes (genus *Sebastes*) led to an incredible diversity of 110 commercially and ecologically important species^57^, only one of which, *Sebastes auriculatus*, the Brown Rockfish, could be discriminated based on the universal 12S MiFish primers used in this study. Species complexes characterized by recent evolutionary radiations such as rockfishes pose challenges for eDNA discrimination. One way to overcome this could be to rely on alternative primers specifically developed for these species. For instance, a rockfish-specific primer set has been developed and successfully discriminated 28 out of 44 commercially harvested species^58^. Further improvements could be also be made through development and curation of more comprehensive DNA reference libraries^59^, or using multiple primers to target different groups.

Traditional survey methods require trained divers that count fish within a restricted area, and thus are expensive and limited in spatial extent. Divers are limited in their ability to see fish by varying water clarity and habitat heterogeneity. We found that single eDNA water samples captured many more taxa than the visual surveys, taxa were discovered much more quickly with additional eDNA samples, and eDNA samples at a given transect had high reproducibility. This suggests that eDNA likely samples a larger effective volume of habitat, integrating biodiversity over small spatial and temporal scales and circumventing difficulties caused by differences in microhabitat types and fish behavior^32,42^. Our results collectively demonstrate the repeatability and reliability of eDNA approaches and efficiency as compared to UVC. Although triplicate eDNA water samples were collected within only an hour of each other, they displayed smaller than expected taxonomic variability and smaller compositional variability as compared to UVC. In addition, fewer eDNA samples were required to detect a given number of taxa in the SBC compared to diver surveys. Lastly, eDNA had increased power to detect spatial correlation in fish communities. On the other hand, eDNA methods have their own limitations that will become clearer as we better understand the spatio-temporal dynamics of DNA molecules in the ocean. PCR amplification bias can also be an issue with eDNA and PCR replicates can be used to alleviate it. The fact that 22 species were detected only through UVC in this study reinforces that UVC and eDNA provide complementary information about biodiversity, and underscores the continued value of traditional visual surveys.

There is growing evidence from freshwater systems^60^, and pioneering studies in marine environments^30^ that eDNA is a promising tool to investigate patterns and change in marine biodiversity. Our results confirm that eDNA can resolve spatial patterns in marine communities even at relatively fine scales, and demonstrate its utility for the measurement of β diversity. A previous study using diver survey data showed that β diversity of kelp forest fish communities in our region mainly arise over broad spatial scales exceeding the extent of the present study rather than small spatial scales^36^. A similar study using eDNA data could reveal much more fine-grained variability in these communities that was undetected by traditional methods. This capability to detect differences at a finer spatial scale would be a more powerful way to guide spatial management, including Marine Protected Areas (MPAs), offshore energy development, or aquaculture. Similarly, eDNA could be used to track changes in fish communities over time, in the face of global climate change and consequently shifting species distributions^61^. Our results significantly bolster the conclusion that eDNA will be an invaluable tool to track spatiotemporal changes in aquatic ecosystems, including coastal oceans. We expect the effective area an eDNA sample integrates to be a function of the rates of water flow, eDNA generation and degradation. Further work is needed to advance our understanding of the dynamics of eDNA in the natural environment, including modeling of how differences in water movement and eDNA decay affect within and between-site variability.

For fish communities, our results show that eDNA can provide better spatial resolution of differences in β diversity than visual surveys, the current standard measurement. These results, combined with the relatively low field effort required to collect eDNA samples and the simultaneously dropping costs and improving methods of DNA metabarcoding, reinforce the recommendations of Ausubel et al.^62^, in particular that biodiversity scientists and monitoring programs should begin sampling eDNA as soon as possible across a wide range of aquatic ecosystems.

## Methods

### Study system

We focused on shallow subtidal rocky reefs from the Santa Barbara Channel located in the northern portion of the Southern California Bight (Fig. 1). Anchored to rocky substrate, giant kelp (*Macrocystis pyrifera*) forms underwater forests that provide habitat for a great variety of species^35^, including many commercially and recreationally important fish species. We focused on nine sites (sites 1–9) spanning an 80 km stretch of mainland and two sites (sites 10–11) located on the northern coast of the Santa Cruz island that bound the Santa Barbara Channel (Fig. 1).

### Underwater visual surveys

Underwater visual surveys (UVC) of fish communities in the Santa Barbara Channel have been conducted annually from 2000 to 2018 by the Santa Barbara Coastal Long Term Ecological Research program (SBC LTER: http://sbc.lternet.edu). Each year, in late July to early August, trained divers performed UVC to record the abundances and sizes of fish taxa within multiple fixed 80 m^2^ transects distributed among the eleven rocky reef sites. Over these 19 years, SBC LTER detected 64 distinct taxa of fishes across 748 transects, including 57 bony fishes and seven elasmobranchs. In 2017, we jointly censused fish taxa and sampled water for DNA metabarcoding at 27 transects distributed across the 11 rocky reef sites (2–3 transects per site; Fig. 1). Size of fish taxa visually recorded was converted to biomass (g dry mass m^−2^) using species-specific relationships. Organisms that were difficult to visually identify to species (e.g. *Gibbonsia* spp.) were lumped into higher taxonomic categories and treated as a single taxon in our analyses. A complete description of the sampling methods, geographic and taxonomic coverage and links to the data and metadata are found in Reed^63^.

### eDNA sampling, extraction and sequencing

Triplicate water samples were collected within an hour, simultaneously with UVC, from July 17th to July 25th at each transect survey in 2017 using a 1 L Nalgene polycarbonate bottle 1 m above bottom at the center of each transect. Within each site, we randomly selected all three replicates from one transect and one replicate at the remaining transects for eDNA analysis. In total, we analyzed 49 water samples, allowing us to hierarchically assess variability within transects, among transects within sites, and among sites.

For each eDNA sample, 1 L of water was filtered onto a 47 mm diameter, 0.22 μm pore size, polyvinylidene difluoride (PVDF) membrane filter (Millipore, USA) and stored at − 80 °C prior to extraction. DNA was extracted using a Qiagen DNeasy Blood and Tissue Kit following the manufacturer’s protocol with some modifications. The complete protocol is published on protocols.io (https://dx.doi.org/10.17504/protocols.io.n2udgew). Metabarcoding of the *12S* mitochondrial rRNA gene was completed using a two-step PCR protocol and the MiFish-U primer set^56^. Samples were analyzed alongside other environmental samples across two sequencing plates as follows. Primary PCR reactions were carried out using 1 μl DNA extract in triplicate with a no-template control (NTC) run for each 96-well plate. Primary PCR primers were as follows, listed in 5′ to 3′ direction: Fluidigm CS1 + **MiFish-U-F** ACACTGACGACATGGTTCTACA **GTCGGTAAAACTCGTGCCAGC** and Fluidigm CS2 + **MiFish-U-R** TACGGTAGCAGAGACTTGGTCT **CATAGTGGGGTATCTAATCCCAGTTTG**. Pooled PCR products were then run through an agarose gel to confirm target amplification and lack of off-target amplification across environmental samples and lack of amplification in NTC. PCR products were then purified and size selected using the Agencourt AMPure XP bead system (Beckman Coulter, USA) and a second agarose gel run to confirm primer removal and retention of target amplicons. A 20 μl aliquot of each purified PCR product was then sent to Research Technology Support Facility (RTSF) Genomics Core at Michigan State University (MSU) for secondary PCR amplification and sequencing. Secondary PCR amplification targeted the CS1/CS2 ends of the primary PCR products and added dual indexed, Illumina compatible adapters with barcodes. Secondary Fluidigm PCR primers listed in 5′ to 3′ direction were: PE1-BC-CS1 (forward): AATGATACGGCGACCACCGAGATCT-[i5-BC(index 2)]-ACACTGACGACATGGTTCTACA and PE2-BC-CS2 (reverse): CAAGCAGAAGACGGCATACGAGAT-[i7-BC(index 1)]-TACGGTAGCAGAGACTTGGTCT. Secondary PCR products were run through an agarose gel to confirm presence of target amplicons and lack of off-target amplification in environmental samples and lack of amplification in NTC. They were then run through Invitrogen SequalPrep Normalization Plate (ThermoFisher Scientific) using the manufacturer’s protocol to create a pooled library. The pooled product was then loaded on a standard MiSeq v2 flow cell and sequenced in a 2 × 250 bp paired end format using a v2 500-cycle MiSeq reagent cartridge. A 10% PhiX spike was added. Primers complementary to the Fluidigm CS1 & CS2 oligomers were added to appropriate wells of the reagent cartridge to server as sequencing and index read primers. Base calling was done by Illumina Real Time Analysis (RTA) v1.18.54 and output of RTA was demultiplexed and converted to FastQ format with Illumina Bcl2fastq v2.18.0. We also amplified two negative extraction controls and three PCR controls and sequenced them in parallel with the 49 samples. Also amplified on each sequencing plate was a set of three PCR no-template controls (NTCs) as well as extraction negative controls which were extracted alongside the environmental samples on the plate (1 × per plate corresponding to these samples).

### Bioinformatics

Illumina MiSeq sequences were processed using a bioinformatic pipeline adapted from the *banzai* pipeline^64^ (https://github.com/jimmyodonnell/banzai/blob/master/banzai.sh), which links together bioinformatic programs through a shell script. Reads were merged through PEAR^65^, quality filtered through VSEARCH^66^, demultiplexed using awk, primers removed through cutadapt^67^, dereplicated, and clustered using swarm with d = 1^68^. Taxonomy was assigned through blastn searches to NCBI GenBank’s non-redundant nucleotide database (nt).

Blast results were filtered using MEGAN6’s lowest common ancestor (LCA) algorithm^69^. Only hits with ≥ 85% sequence identity, ≥ 100 bitscore and whose bitscores were within the top 2% of the highest bitscore value for each Operational Taxonomic Unit (OTU) were considered by MEGAN6. The MEGAN6 parameter LCA percent was set to 85, allowing for up to 15% of top hits to be off target and still have the majority taxonomy assigned. This parameter value was chosen to allow for minor numbers of incorrectly annotated GenBank entries—effectively allowing for OTUs which had many high-quality hits to a taxa to still be assigned to that taxa even if there existed a high-bitscore hit to another GenBank sequence annotated to an unrelated taxa. We decided this was more advantageous than the disadvantage caused by ignoring small numbers of true closely related sequences. Furthermore, post-MEGAN6 filtering was performed to ensure only contigs with a hit of ≥ 97% sequence identity and ≥ 300 bitscore were annotated to the species level. Only contigs with a hit of ≥ 95% sequence identity and ≥ 200 bitscore were annotated to the genus level. Annotations were elevated to the next highest taxonomic level for contigs that failed those conditions.

Two studies previously used eDNA to survey rocky reef communities^27,28^. Both studies used a higher percent identity threshold for all blastn hits and were therefore more stringent in their taxonomic assignments. Port et al.^27^ used a threshold of 98% and Andruszkiewicz et al.^28^ used a threshold of 97% identity. We used a similarly high threshold of 97% identity to assign OTUs to the species level and 95% identity to the genus level, but allowed family and higher-level annotations to occur with blast hits above an 85% identity threshold. Since these hits also have MEGAN6’s LCA algorithm applied to them, we can have increased confidence in their accuracy despite their lower percent identity.

Post-bioinformatic processing, samples contained a total number of reads ranging from a maximum of 22,013 reads to a minimum of 1413 reads across a total of 2386 OTUs. This corresponded to a total of 294,010 Eukaryotic reads, 1493 Bacterial reads, and 35,822 reads that were unassigned. Raw FASTQ files contained an increased number of bacterial sequences which have a longer amplification fragment (~ 300 bp) but these reads were largely removed within the bioinformatic pipeline by setting PEAR parameters to limit paired-end read merging assembly to between 100 and 260 bp in length.

Across our PCR no-template controls (NTCs), we detected a total of 9 families that had greater than 5 reads within a NTC sample. This included 8156 reads of *Oncorhynchus kisutch* (Coho Salmon), 379 reads of family *Pleuronectidae* and 143 reads of *Homo sapiens*. These OTUs were dropped from our analysis. The remaining detections had fewer than 50 reads across 6 NTCs and included *Engraulidae* (22 reads), *Paralichthyidae* (43 reads), *Sebastidae* (30 reads), *Clupeidae* (13 reads), *Bovidae* (15 reads), and *Suidae* (8 reads). Terrestrial taxa (*Bovidae* and *Suidae*) were removed across our analysis but for the remaining taxa we considered this contamination minor and acceptable in sequenced NTCs. Across two extraction blanks which were sequenced, the vast majority of reads were to *Homo sapiens* (18,951 total, due to one sample with 18,944 reads). Remaining contamination was < 5 reads total per taxa apart from 17 reads of *Oncorhynchus kisutch* and 15 reads of *Paralichthyidae* which we considered minor and acceptable in sequenced extraction blanks.

### Defining the regional pool of taxa

We selected 1360 and 39 OTUs assigned to either bony (*Actinopteri*) and cartilaginous (*Chondrichthyes*) fishes. These two classes exhibited a total of 239,818 and 5472 sequence reads across all samples respectively. We removed 9 OTUs corresponding to taxa whose geographic distribution did not overlap with our study area as well as 58 OTUs assigned at a taxonomic level higher than family. We then merged OTUs at the species levels resulting in 118 and 19 OTUs for *Actinopteri* and *Chondrichthyes* respectively, from which we removed 20 OTUs exhibiting fewer than five reads across all samples. We also decided to hierarchically merge annotations for OTUs un-assigned at species or genus levels in order to better compare data types between eDNA and UVC and conservatively estimate the number of unique taxonomies detected through eDNA. This accounted for the potential inflation of unique taxonomic annotations in our sequence data caused by minor sequence variation within a species or by small errors in the sequencing process. A typical example would be when two closely related OTUs share a genus level annotation, but while one highly abundant OTU is assigned to a species, the other less abundant OTU is left unassigned at the species level. Our assumption is that the unassigned OTU is likely the result of sequence error or minor sequence variation within a species that makes it a worse match to the reference sequence. Here we are conservatively measuring diversity by merging these two OTUs and assigning their taxonomy to the most abundant one. This reduces our ability to detect a closely related species not found within our reference database but for the purpose of this study we considered annotation errors caused by sequence variation from a dominating organism were more detrimental in our dataset than losing sequence variation caused from detecting a mixture of species absent and present within our reference database. This leads us to conservatively estimate total diversity detected by our sequence data. We hierarchically merged at both the genus and family levels in the presence of unassigned OTUs. When multiple OTUs were assigned to the same genus and one was unassigned at the species level we did as follows: (i) if an OTU unassigned at the species level was most abundant, we merged and assigned all OTU(s) to the genus-level (N = 2; both *Sebastes* genus) or (ii) if one of the species-level OTUs was most abundant, we merged and assigned the genus-level OTU to that species-level OTU annotation (N = 24). We adopted the same strategy with families containing OTUs unassigned at the genus level as follows: (i) if the OTU assigned at the family-level was most abundant, we merged and assigned all OTU(s) to the family-level (N = 4) and (ii) if a genus- (or species-) level OTU was most abundant, we merged and assigned the family level OTU to that OTU's taxonomy (N = 18). This procedure allowed us to define the regional pool of taxa in a more equivalent way to the visual data, narrowing down the final number of unique taxonomically assigned OTUs to 69. For comparison purpose, we also produced a table of 1402 OTUs for which we limited OTUs to the phylum Chordata and only removed OTUs from common contaminants (families *Hominidae*, *Bovidae*, *Canidae*, *Suidae*), birds (Class *Aves*), and fish familes not present in this geographic area (families *Centrarchidae*, *Cichlidae*, *Terapontidae*), but did not follow any other of the previously mentioned filtering steps. Finally, samples were rarefied to the lowest read number (N = 829) using the program phyloseq^70^ in R v3.6.0.

### Statistical analysis

We additively partitioned^71^ the regional taxonomic richness estimated with eDNA (${\gamma }_{eDNA}$) into four hierarchical spatial components as:

1 $$ \gamma _{{eDNA}}  = \alpha _{{BOT}}  + \beta _{{BOT}}  + \beta _{{TR}}  + \beta _{{SITE}} . $$

${\alpha }_{BOT}$ corresponds to the mean taxonomic richness within individual bottles. The three $\beta$ components represent the mean spatial variability among samples within a given hierarchical level, that is variability among triplicate bottles within transects (${\beta }_{BOT}$), variability among transects within sites (${\beta }_{TR}$) and variability among sites within the Santa Barbara Channel (${\beta }_{SITE}$). We similarly partitioned the taxonomic richness estimated with UVC ($\gamma _{{UVC}}$) into three hierarchical spatial components as:

2 $$ \gamma _{{UVC}}  = \alpha _{{TR}}  + \beta _{{TR}}  + \beta _{{SITE}} . $$

${\alpha }_{TR}$ corresponds to the mean taxonomic richness within transects. The smallest hierarchical level being different for eDNA and UVC (bottle vs. transect), ${\alpha }_{BOT}+{\beta }_{BOT}$ in Eq. (1) equals ${\alpha }_{TR}$ in Eq. (2). We then evaluated the significance of each component using randomization of the original sample by taxa matrix. We compared each component to its expected value calculated 999 times by individual based randomization of the original sample by taxa matrix. We built Taxa-Area Relationships (TAR) by randomly selecting an initial sample (i.e., a bottle for eDNA or a transect for UVC), and then increasing the number of samples by one unit by including neighboring samples. This procedure was repeated for all possible combination of initial sample to draw 95% Confidence Intervals (CI), from one up to 49 water samples for eDNA or 27 transects for UVC. We then investigated the relationship between taxa richness and area sampled by fitting different models. We compared linear (y = a + b × S), exponential (y = a × e^b×S^) or piecewise-linear relationships (two linear models with one breaking point^72^) between taxonomic richness (y) and area sampled (S). In the case of exponential relationships, the steeper the slope (b) the higher the spatial turnover in taxonomic richness. Since historical records of fish communities in the Santa Barbara Channel were available across 748 transects surveyed from 2000 to 2018, we used this information to estimate the TAR for UVC beyond the 27 focal transects. Additional transects were randomly drawn from the historical records, and 499 bootstraps were performed to estimate 95% CI.

We computed the Bray–Curtis dissimilarity between pairs of samples to evaluate spatial variation in community structure. We used variation partitioning to hierarchically partition the total variation in community structure among samples into the following spatial components: variation among sites, among transects and among triplicates water samples for eDNA^73^. Geographic distances among samples ranged from 0 (triplicate samples) to 74 km for eDNA or to 14 m to 74 km for UVC. We examined the relationship between ecological similarity, measured as (1 − *D*) with *D* the Bray–Curtis dissimilarity index, and geographic distance between pairs of samples and tested the significance of these relationships based on Mantel’s tests. We also performed a power analysis of Mantel’s test at a significance level of 0.05 to draw power curves for eDNA and UVC for effect sizes ranging from 0 to 0.5^74^. We then performed Non-Metric Multidimensional Scaling (NMDS) based on the Bray–Curtis dissimilarities to visualize compositional differences among samples. Finally, we conducted indicator species analysis following Dufrene and Legendre^75^ to identify taxa associated with specific rocky reef sites. All Bray–Curtis dissimilarities were based on the square root number of reads for eDNA and observed biomass for UVC of fish taxa. All analyses were performed using R version 3.6.0^76^. Additive diversity partitioning was performed using the *adipart* function of the vegan package^77^ and randomizations of the original data were carried out using the *oecosimu* function from vegan. Piecewise-linear relationships were build using the R package *segmented*^72^. Power analysis of Mantel’s test was performed using the function *mantelPower* from R package *biotools*^74^.

## Supplementary Information

Supplementary Information.

## Data Availability

Environmental DNA data has been archived in the Sequence Read Archive (SRA) database (https://www.ncbi.nlm.nih.gov/sra) under the accession number PRJNA667508.
