# Supplementary material for: Environmental DNA reveals the fine-grained and hierarchical spatial structure of kelp forest fish communities
Source: Sci Rep. 2021 Jul 14;11:14439. doi: 10.1038/s41598-021-93859-5 (PMC8280230; doi:10.1038/s41598-021-93859-5)

Supplementary Information to "*Environmental DNA reveals the fine-grained and hierarchical spatial structure of kelp forest fish communities*". Thomas Lamy, Kathleen J. Pitz, Francisco P. Chavez, Christie E. Yorke and Robert J. Miller.

Table SM1. List of the 38 taxa detected using Underwater Visual Census (UVC). For each taxon we indicate its relative biomass (**%**) and total number of individuals (**N**) observed across all surveyed transects (N = 27). When a taxon was also detected using eDNA metabarcoding, the corresponding taxonomic level (**eDNA**) and the number of reads across all samples (**Reads**) are provided. For instance, nine taxa of rockfishes (*Sebastes* spp.) were lumped at the genus level to match detection of the genus *Sebastes* using eDNA metabarcoding. eDNA metabarcoding detected 3,220 sequence reads of *Sebastes* spp. across all samples. "*No reference*" indicates the absence of reference sequences in GenBank.
[1]No reference at the species level; [2]eDNA sequence found but only 4 reads; [3]303 reads but merged at the genus level; [4]no reference at the species and genus level; [5]eDNA sequence found but only 3 reads.

Order	Family	Taxa	%	N	eDNA	Reads
Perciformes	Clinidae	*Gibbonsia* spp. (Crevice Kelpfish)	0.054	8	Species	3179
Perciformes	Embiotocidae	*Brachyistius frenatus* (Kelp Surfperch)	0.034	13	Species	2581
Perciformes	Embiotocidae	*Embiotoca jacksoni* (Black Surfperch)	8.010	71	Species	23895
Perciformes	Embiotocidae	*Rhacochilus vacca* (Pile Surfperch)	0.883	9	Species	3076
Perciformes	Kyphosidae	*Medialuna californiensis* (Halfmoon)	7.164	15	Species	6605
Perciformes	Labridae	*Oxyjulis californica* (Senorita)	2.383	189	Species	37875
Perciformes	Labridae	*Semicossyphus pulcher* (California Sheephead)	18.506	46	Species	14228
Perciformes	Pomacentridae	*Chromis punctipinnis* (Blacksmith)	1.191	118	Species	23050
Perciformes	Pomacentridae	*Hypsypops rubicundus* (Garibaldi)	5.118	19	Species	1600
Pleuronectiformes	Paralichthyidae	*Citharichthys stigmaeus* (Speckled Sanddab)	0.034	21	Species	1872
Scorpaeniformes	Cottidae	*Scorpaenichthys marmoratus* (Cabezon)	0.353	7	Species	1502
Scorpaeniformes	Hexagrammidae	*Oxylebius pictus* (Painted Greenling)	0.121	16	Species	519
Pleuronectiformes	Paralichthyidae	*Paralichthys californicus* (California Halibut)	0.464	1	Genus[1]	783
Scorpaeniformes	Sebastidae	*Sebastes* spp. (Rockfish)	0.008	4	Genus	3220
Scorpaeniformes	Sebastidae	*Sebastes atrovirens* (Kelp Rockfish)	0.441	5	Genus	3220
Scorpaeniformes	Sebastidae	*Sebastes carnatus* (Gopher Rockfish)	0.532	2	Genus	3220
Scorpaeniformes	Sebastidae	*Sebastes caurinus* (Copper Rockfish)	0.133	1	Genus	3220
Scorpaeniformes	Sebastidae	*Sebastes mystinus* (Blue Rockfish)	0.072	1	Genus[2]	3220
Scorpaeniformes	Sebastidae	*Sebastes paucispinis* (Boccacio)	0.007	6	Genus[3]	3220

Scorpaeniformes	Sebastidae	*Sebastes rastrelliger* (Grass Rockfish)	0.357	3	Genus	3220
Scorpaeniformes	Sebastidae	*Sebastes serranoides* (Olive Rockfish)	0.453	5	Genus	3220
Scorpaeniformes	Sebastidae	*Sebastes serriceps* (Treefish)	0.019	1	Genus	3220
Squatiniformes	Squatinidae	*Squatina californica* (Pacific Angel Shark)	19.720	2	Genus	877
Perciformes	Gobiidae	*Lythrypnus dalli* (Bluebanded Goby)	0.246	441	Family	142
Perciformes	Gobiidae	*Rhinogobiops nicholsii* (Blackeye Goby)	0.001	2	Family[4]	142
Perciformes	Haemulidae	*Anisotremus davidsonii* (Sargo)	0.935	30	Family[4]	1198
Perciformes	Labrisomidae	*Alloclinus holderi* (Island Kelpfish)	0.004	5	Family[4]	685
Batrachoidiformes	Batrachoididae	*Porichthys notatus* (Plainfin Midshipman)	0.104	1	NO[5]	0
Perciformes	Chaenopsidae	*Neoclinus blanchardi* (Sarcastic Fringehead)	0.000	1	NO	0
Perciformes	Kyphosidae	*Girella nigricans* (Opaleye)	4.356	8	NO	0
Perciformes	Labridae	*Halichoeres semicinctus* (Rock Wrasse)	1.660	37	NO	0
Perciformes	Serranidae	*Paralabrax clathratus* (Kelp Bass)	18.236	85	NO	0
Perciformes	Serranidae	*Paralabrax nebulifer* (Barred Sandbass)	4.497	10	NO	0
Perciformes	Embiotocidae	*Phanerodon furcatus* (White Surfperch)	0.094	4	No sequence	0
Pleuronectiformes	Bothidae	Bothidae (Flatfish)	0.019	1	No sequence	0
Scorpaeniformes	Cottidae	Cottidae (Sculpin)	0.008	5	No sequence	0
Scorpaeniformes	Cottidae	*Leiocottus hirundo* (Lavender Sculpin)	0.001	1	No sequence	0
Perciformes	Embiotocidae	*Rhacochilus toxotes* (Rubberlip Surfperch)	3.782	6	No sequence	0

Table SM2. List of the 51 taxa only detected with eDNA metabarcoding. For each taxon we provide the total number of sequence reads across all samples (**Reads**) and their taxonomic level (**Level**). **LTER** indicates whether a taxon was visually detected by SBC LTER from 2000 to 2018 across 748 transects although the taxon was not visually detected across the 27 compared transects. **Pelagic** distinguishes pelagic *versus* benthic taxa. [1]species level in UVC but family level for eDNA.

Order	Family	Taxa	Reads	Level	LTER	Pelagic
Blenniiformes	Clinidae	*Heterostichus rostratus* (Giant Kelpfish)	2528	Species	YES	NO
Carcharhiniformes	Triakidae	*Triakis semifasciata* (Leopard Shark)	445	Species	YES	NO
Heterodontiformes	Heterodontidae	*Heterodontus francisci* (Horn Shark)	366	Species	YES	NO
Myliobatiformes	Myliobatidae	*Myliobatis californica* (Bat Ray)	2910	Species	YES	NO
Perciformes	Hexagrammidae	*Ophiodon elongatus* (Lingcod)	101	Species	YES	NO
Syngnathiformes	Syngnathidae	*Syngnathus leptorhynchus* (Bay Pipefish)	1097	Species	YES	NO
unknown	Embiotocidae	*Cymatogaster aggregata* (Barred surfperch)	555	Species	YES	NO
Anguilliformes	Muraenidae	Muraenidae (Moray eels)	1771	Family	YES[1]	NO
Carcharhiniformes	Scyliorhinidae	Scyliorhinidae (Catsharks)	591	Family	YES	NO
Rhinopristiformes	Rhinobatidae	Rhinobatidae (Shovelnose Guitarfish)	25	Family	YES[1]	NO
unknown	Sphyraenidae	Sphyraenidae (Barracuda)	1916	Family	YES[1]	YES
Aulopiformes	Synodontidae	*Synodus lucioceps* (California lizardfish)	933	Species	NO	NO
Blenniiformes	Blenniidae	*Hypsoblennius gilberti* (Rockpool blenny)	2377	Species	NO	NO
Carangiformes	Carangidae	*Trachurus symmetricus* (Californian jack mackerel)	4455	Species	NO	YES
Clupeiformes	Clupeidae	*Sardinops melanostictus* (Japanese pilchard)	60171	Species	NO	YES
Clupeiformes	Engraulidae	*Engraulis mordax* (Californian anchovy)	2582	Species	NO	YES
Hexanchiformes	Hexanchidae	*Notorynchus cepedianus* (Broadnose sevengill shark)	149	Species	NO	NO
Lamniformes	Alopiidae	*Alopias vulpinus* (Common thresher)	27	Species	NO	YES
Lamniformes	Alopiidae	*Carcharodon carcharias* (Great white shark)	11	Species	NO	YES
Ophidiiformes	Ophidiidae	*Chilara taylori* (Spotted cusk eel)	49	Species	NO	NO
Perciformes	Agonidae	*Odontopyxis trispinosa* (Pygmy poacher)	105	Species	NO	NO
Perciformes	Cottidae	*Artedius lateralis* (Smoothhead sculpin)	46	Species	NO	NO

Perciformes	Liparidae	*Liparis mucosus* (Slimy snailfish)	433	Species	NO	NO
Perciformes	Scorpaenidae	*Scorpaenodes evides* (Cheekspot scorpionfish)	760	Species	NO	NO
Pleuronectiformes	Paralichthyidae	*Xystreurys liolepis* (Fantail flounder)	175	Species	NO	NO
Pleuronectiformes	Paralichthyidae	*Citharichthys sordidus* (Pacific sanddab)	15	Species	NO	NO
Pleuronectiformes	Pleuronectidae	*Pleuronichthys coenosus* (C-O sole)	352	Species	NO	NO
Pleuronectiformes	Pleuronectidae	*Pleuronichthys decurrens* (Curlfin sole)	177	Species	NO	NO
Pleuronectiformes	Pleuronectidae	*Lyopsetta exilis* (Slender sole)	8	Species	NO	NO
Rajiformes	Rajidae	Raja inornata (California skate)	9	Species	NO	NO
Salmoniformes	Salmonidae	*Salmo salar* (Atlantic salmon)	13	Species	NO	YES
Scombriformes	Stromateidae	*Peprilus medius* (Pacific harvestfish)	86	Species	NO	YES
unknown	Embiotocidae	*Amphistichus argenteus* (Barred surfperch)	1851	Species	NO	NO
Carcharhiniformes	Triakidae	*Mustelus* spp. (Smooth-hound)	22	Genus	NO	NO
Perciformes	Cottidae	*Icelinus* spp. (Sculpin)	14	Genus	NO	NO
Perciformes	Zaniolepididae	*Zaniolepis* spp. (Combfish)	71	Genus	NO	NO
Pleuronectiformes	Paralichthyidae	*Etropus* spp. (Large-tooth flounders)	9	Genus	NO	NO
Scombriformes	Scombridae	*Scomber* spp. (Mackerels)	2528	Genus	NO	YES
Atheriniformes	Atherinopsidae	Atherinopsidae (Neotropical silversides)	19407	Family	NO	YES
Beloniformes	Exocoetidae	Exocoetidae (Flying fish)	102	Family	NO	YES
Blenniiformes	Gobiesocidae	Gobiesocidae (Clingfishes)	2153	Family	NO	NO
Ephippiformes	Ephippidae	Ephippidae (Spadefishes)	6	Family	NO	NO
Lutjaniformes	Lutjanidae	Lutjanidae (Snapper)	46	Family	NO	NO
Myctophiformes	Myctophidae	Myctophidae (Lanternfish)	5	Family	NO	YES
Myliobatiformes	Urotrygonidae	Urotrygonidae (American round stingrays)	17	Family	NO	NO
Perciformes	Pholidae	Pholidae (Gunnels)	43	Family	NO	NO
Perciformes	Stichaeidae	Stichaeidae (Pricklebacks)	27	Family	NO	NO
unknown	Opistognathidae	Opistognathidae (jawfishes)	76	Family	NO	NO
unknown	Sciaenidae	Sciaenidae (Drums or croakers)	4489	Family	NO	NO
Uranoscopiformes	Ammodytidae	Ammodytidae (Sand lance)	22	Family	NO	NO
unknown	Platyrhinidae	Platyrhinidae (Thornbacks)	11	Family	NO	NO

Figure SM1. The 18 taxa jointly detected by eDNA and UVC. (A) Relationship between the mean number of reads per bottle and the mean taxa biomass per transect on a log-log scale. (B) Relative number of reads and biomass of these taxa averaged across the 11 sites.

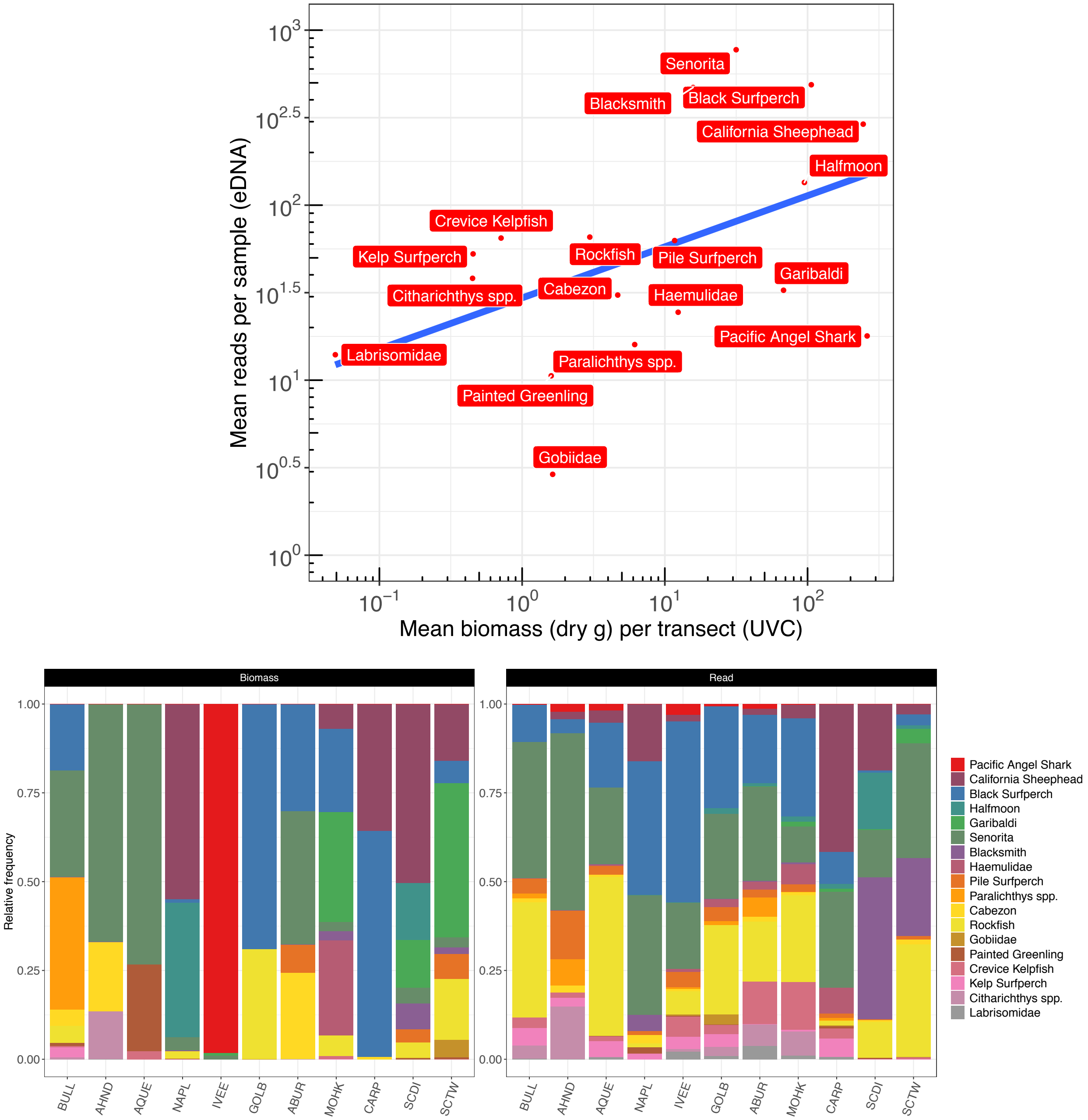

Figure SM2. Distribution of the 80 taxa detected in this study across the 11 sites. The mean observed biomass (g) and number of reads per site is provided for each taxon. Mean values were standardized by their maximum value and log-transformed for comparison. Sites are ordered by their number.

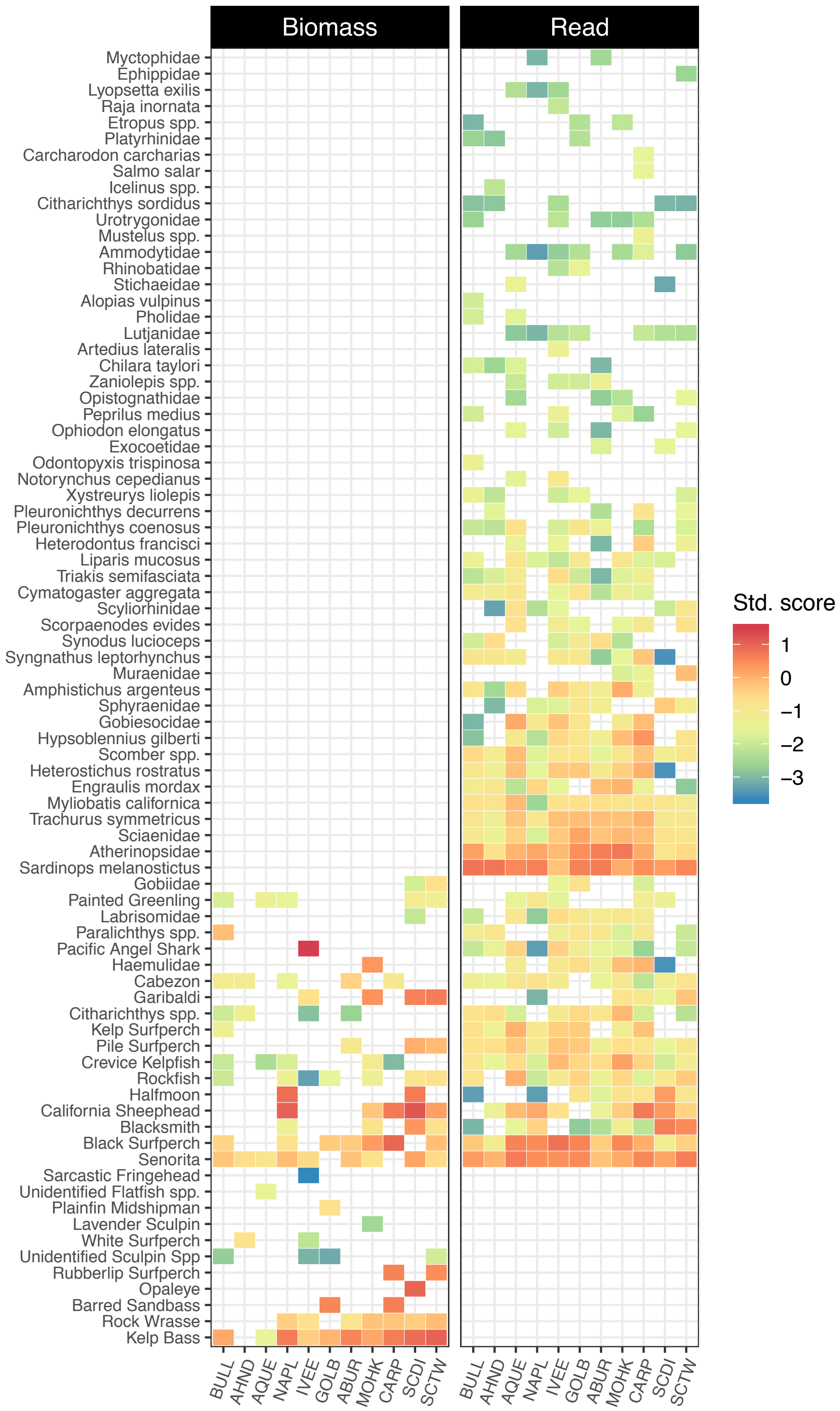

Figure SM3. Mean observed biomass per site divided into the contribution of the 10 taxa not detected using eDNA (color code) and the merged biomass of the 27 taxa detected by eDNA (in black).

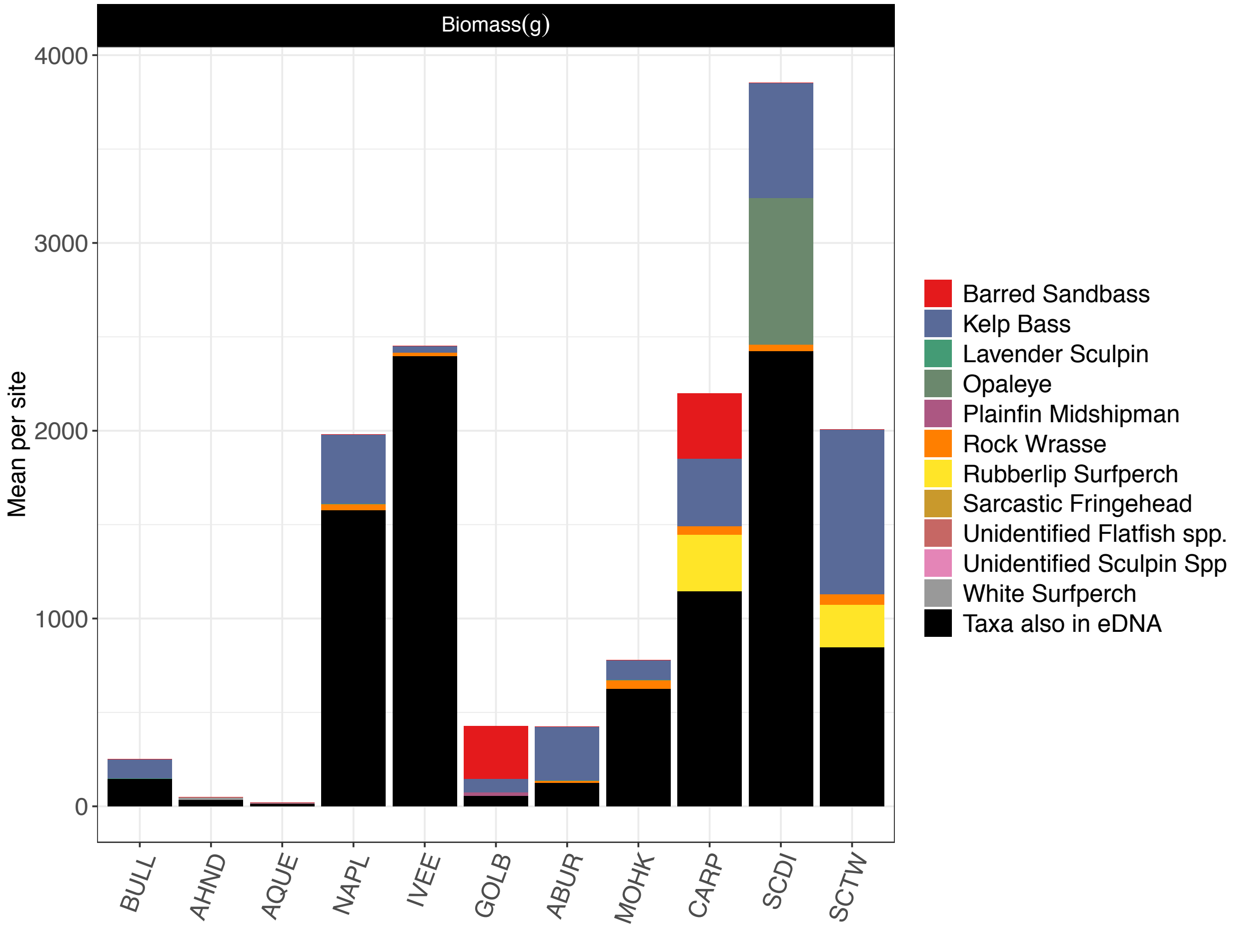

Figure SM4. Mean number of reads per site divided into the contribution of the 51 taxa not detected using UVC (color code) and the merged number of reads for the 18 taxa detected by UVC (in black).

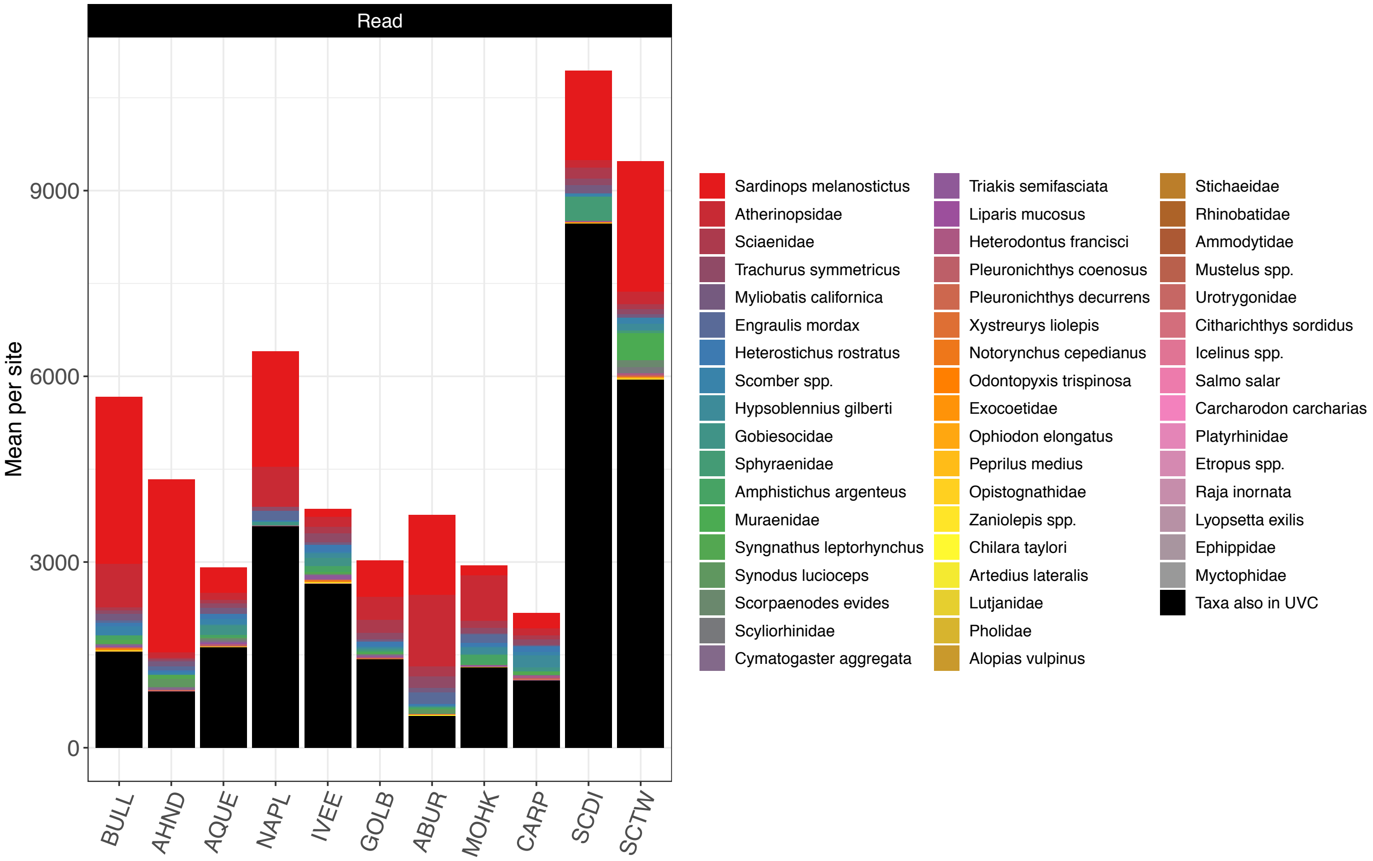

Figure SM5. Taxa accumulation curves estimating how many more taxa could have been detected using UVC if more transects were sampled by randomly selecting additional transects surveyed from 2000 to 2018 by SBC LTER (black line). 64 distinct fish taxa were detected during that time. Results suggest 69 transects are needed to detected at least 50 taxa. Blue and red lines correspond to the taxa accumulation curves with space eDNA and UVC as shown in Figure 3.

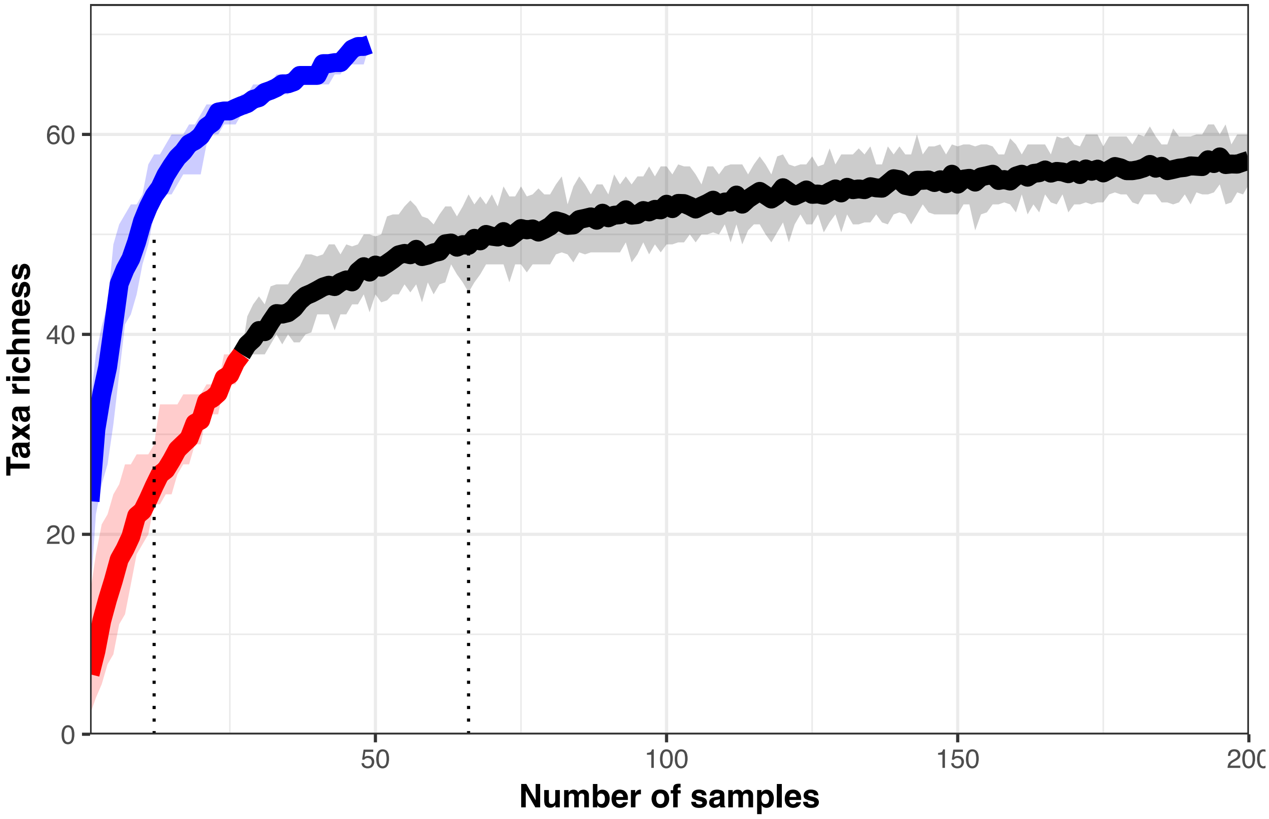

Figure SM6. Taxa scores for the Non-Metric Multidimensional Scaling (NMDS) ordinations for eDNA and UVC (See figure 5). Taxa jointly detected by both methods are shown in red while taxa unique to each method are shown in black. Indicator species are shown within boxes.

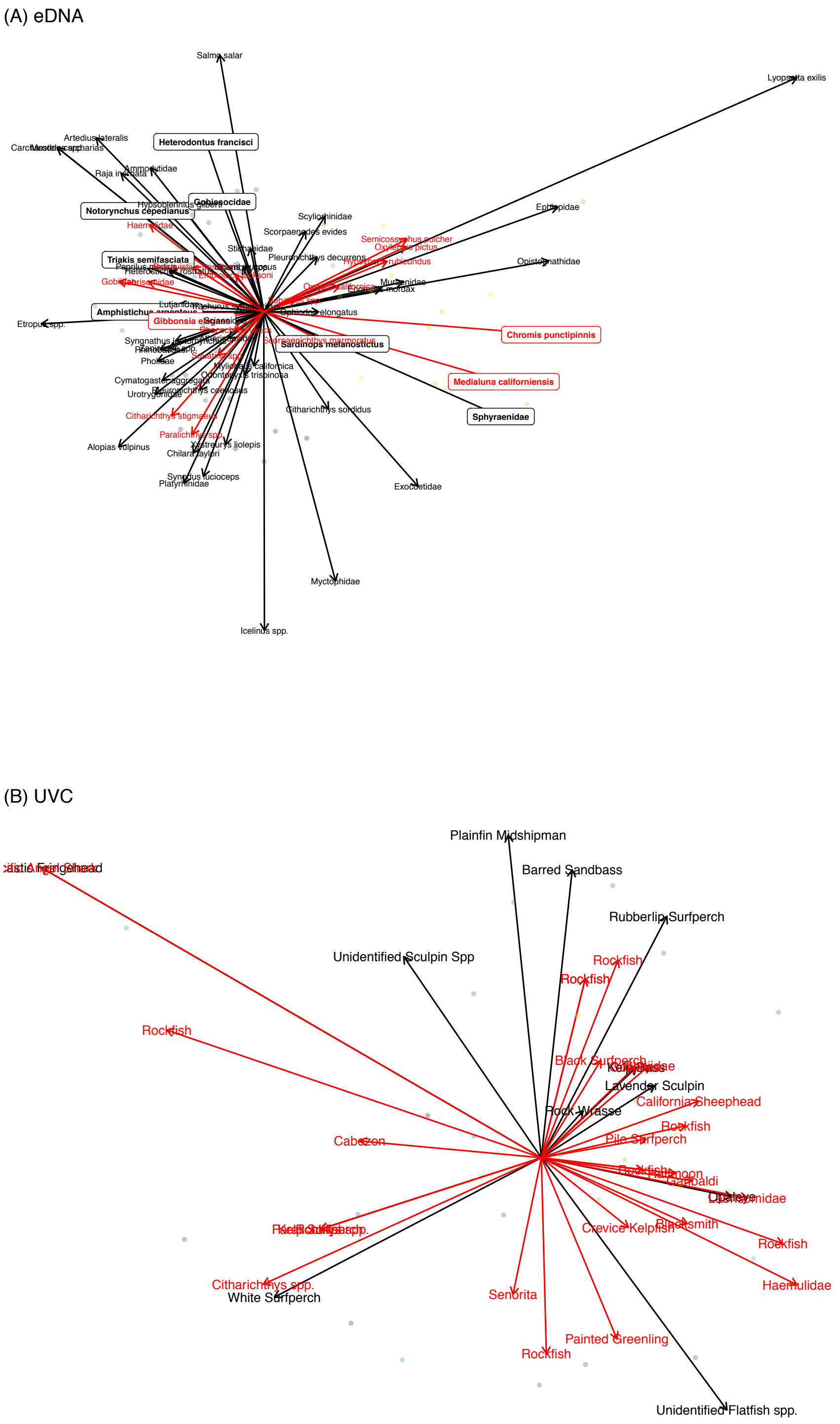

Supplement: Supplementary file 1 — Supplementary Information. [file 41598_2021_93859_MOESM1_ESM.pdf]
